# Incidence of Midfoot Instability Associated With Medial Malleolus Fractures: A Retrospective Cohort Study

**DOI:** 10.7759/cureus.102100

**Published:** 2026-01-22

**Authors:** Zain Al Abdeen Al Zuabi, Eva R Gil Monzó, Velayudhan b Kiliyanpilakkil, Musammad r Begum, Bianca d Chua, Hesham m Youssef, Wai Wai w Mar, Mohamed H Gad, Natalie b Marzouqa, Chandra S Pasapula

**Affiliations:** 1 Medicine and Surgery, The Queen Elizabeth Hospital King's Lynn NHS Foundation Trust, King's Lynn, GBR; 2 Orthopaedics, Hospital Universitario Doctor Peset, Valencia, ESP; 3 Orthopaedics and Trauma, The Queen Elizabeth Hospital King's Lynn NHS Foundation Trust, King's Lynn, GBR; 4 Trauma and Orthopaedics, Broomfield Hospital, Chelmsford, GBR; 5 Urology, University College Hospital, London, GBR; 6 Trauma and Orthopaedics, Norfolk and Norwich University Hospitals NHS Foundation Trust, Norwich, GBR; 7 Orthopaedics and Trauma, Antrim Area Hospital, Antrim, GBR; 8 Medicine and Surgery, Guy's and St Thomas' NHS Foundation Trust, London, GBR; 9 General Surgery, Causeway Hospital, Coleraine, GBR

**Keywords:** cohort study, first ray instability, medial malleolus, midfoot instability, talonavicular laxity

## Abstract

Background: The incidence of secondary medial arch instability (talonavicular and first ray instability) associated with medial malleolus fractures has not been totally quantified, and to date, its contribution to poor foot function is unknown.

Methods: Twenty-five patients with various mechanisms of ankle injuries associated with medial malleolus fractures who underwent surgical treatment were assessed for patient demographics, mechanism of injury, type of fracture and quantity of medial malleolus displacement in X-rays prior to surgery, type of fixation, and level of midfoot instability, assessed by determining and quantifying talonavicular laxity and first ray instability.

Results: In rotational ankle fractures with the medial malleolus involved, increased midfoot laxity and first ray instability were present in injured ankles compared to the uninjured (p<0.05). In non-rotational ankle fractures (supination adduction/supination plantar flexion), affected feet also had a mean increase in lateral translation scores and first ray instability scores but without statistical significance (p>0.05). There was no significant difference in the spring ligament thickness between injured and uninjured feet (p>0.05), and the displacement of the medial malleolus was not a predictor for the development of increased talonavicular laxity (p>0.05).

Conclusion: Ankle fractures with medial malleolus fractures have a significantly higher incidence of medial arch instability. Rotational ankle injuries, arising from supination external rotation and pronation injuries, can significantly affect medial arch stability. We advocate that surgical restoration of superficial deltoid-spring/capsular-ligamentous integrity and/or early post-operative orthotics after medial malleolar fractures may protect the first ray destabilization and preserve future foot function.

## Introduction

Ankle fractures are a common occurrence [1,2]. Outcomes can be variable, with some authors reporting poor outcomes after surgical treatment [3]. Verhage et al. and Stufkens et al. found that ankle fractures that involve the medial malleolus, even if isolated, had worse outcomes [4,5]. Ankle fractures with posterior malleolus fractures have also been shown to have worse outcomes [6]. Blom demonstrated that posteromedial fracture extension had worse outcomes [7], reflecting wider capsuloligamentous disruption.

Tejwani compared patients with bimalleolar fractures to those with lateral malleolus and deltoid rupture and found worse outcomes at 12 months in the former [8]. Increased talonavicular (TN)/spring ligament (SL) laxity has been demonstrated in fractures with acute deltoid ligament (DL) ruptures, which subsequently develop type 1 first ray instability (FRI) [9,10]. Fukuyama also demonstrated that 50% of patients with bimalleolar ankle fractures had a deep deltoid injury [11].

The superficial deltoid and SL are interconnected. Disruption of one affects the function of the other. Disruption of the superficial deltoid (tibiospring), capsule or direct injury/strain to the SL from pronatory injury forces may induce TN laxity and secondary type 1 FRI that develops after medial malleolus fractures. To date, this has not been quantified, and therefore, its potential contribution to poor foot function is not known [12,13]. 

The aims of this study are, firstly, to quantify the increased medial arch instability in patients who had medial malleolar fractures compared to the non-injured side and how different injury types (rotational and non-rotational fractures) affect medial arch stability, and , secondly, the evaluation of the SL's thickness between injured and uninjured feet and whether the medial malleolus fracture and its initial displacement in preoperative rays is a predictor of medial instability.

## Materials and methods

Ethical approval was obtained for this study (West of Scotland REC 21/WS/0164). Twenty-five patients consented to this study. Clinical data, body mass index (BMI), Beighton score (scored 1-9, a greater score means more significant laxity, and if it was > 5, was used to differentiate generalised laxity from local laxity) and foot length are recorded in Table 1.

**Table 1:** Patients' demographics and mechanism of injury (according to Lauge-Hansen classification).

Patient No	Gender	Age (year)	BMI	Beighton score	Foot length (cm)		Time since injury assessment (weeks)	Type of fracture configuration	Laterality of injury
					R	L			
1	Female	46	24	03-Sep	23.5cm	23.5cm	40 weeks	SER 4	Left
2	Female	55	29.1	01-Sep	24	24	33 weeks	PER 4	Left
3	Female	69	26.5	01-Sep	24cm	24cm	64 weeks	SER 4	Right
4	Female	61	34.6	03-Sep	23cm	23cm	96 weeks	SER 4	Left
5	Female	59	35.7	03-Sep	25cm	24.5cm	76 weeks	SER 4 /medial vertical shear	Left
6	Female	40	29.5	02-Sep	30cm	30cm	80 weeks	SER 4	Left
7	Male	55	32.5	01-Sep	26.5cm	26.5cm	48 weeks	SER 4	Right
8	Female	63	32.2	02-Sep	25cm	25cm	28 weeks	SER 4	Left
9	Female	42	34.5	04-Sep	24.5cm	24.5cm	52 weeks	SER 4	Right
10	Female	70	36	N/A	24cm	24cm	28 weeks	SER 4	Left
11	Female	54	25.5	04-Sep	25cm	25cm	62 weeks	SER 4	Left
12	Female	63	3.6	0-9	25cm	25cm	76 weeks	SER 4/medial vertical shear	Right
13	Male	18	25.7	02-Sep	24.5cm	24.5cm	64 weeks	PER 4	Right
14	Female	76	39.1	01-Sep	23cm	23cm	72 weeks	SER 4	Left
										15	Female	48	44.1	02-Sep	22.5cm	23cm	84 weeks	SA 1	Right
16	Female	58	29.8	01-Sep	23.5cm	23.0cm	90 weeks	SER 4	Left										
										17	Male	66	27.4	01-Sep	27cm	28cm	89 weeks	PA 3	Right
18	Female	76	27.9	02-Sep	24cm	23cm	205 weeks	SA 2	Right										
										19	Female	39	31.1	05-Sep	23.5cm	23.5cm	236 weeks	SA 1	Left
20	Male	67	24.8	01-Sep	26cm	26cm	75 weeks	PE 1	Right
21	Female	55	29	02-Sep	25.0cm	25.0cm	21 weeks	SER 4	Right
										22	Female	57	27.2	02-Sep	26cm	26cm	17 weeks	SER 4	Left
23	Female	48	28.8	02-Sep	25cm	25cm	36 weeks	Posterior pilon fracture and coronal fracture in the medial malleolus	Left										
										24	Female	62	33.4	03-Sep	26cm	26cm	22 weeks	SER 4	Left
25	Female	33	29	02-Sep	25.5cm	25.5cm	7 weeks	PER 4	Left										

Inclusion criteria included all patients with ankle fractures associated with medial malleolar fractures who underwent surgical treatment between 2021 and 2022. Radiographs identified fracture types (including posterior malleolar) based on the Lauge-Hansen classification. In all patients, the displacement of talus medial clear on AP X-rays was measured preoperatively. Those that did not fall into these subtypes were descriptively classified. The quality and type of fixation were assessed (Table 2).

**Table 2:** Number of patients, injury type of fracture and fracture fixation: lateral, medial and posterior.

Patient no	Type of fracture configuration	Displacement of talus medial on AP (preoperatory X-ray)	Fracture morphology/comminution of the medial malleolus	Lateral side	Posterior malleolus involved	Lateral fixation	Medial fixation	Posterior fixation
1	SER 4	Medial displacement: 5mm posteriorly subluxed	Transverse	Oblique, Weber B	Yes	Lag screw and neutralization plate	2 screws	-
2	PER 4	Medial displacement: 3 mm	Vertical shear	Oblique long above the syndesmosis	Yes	1 neutralisation plate	Antiglide plate and 1 lag screw	-
3	SER 4	Medial displacement: 7 mm	Transverse	Oblique comminuted	Yes	Lag screw and neutralization plate	2 screws	-
4	SER 4	Medial displacement: 10mm	Transverse	Oblique Weber B	NO	Lag screw and neutralization plate	1 screw	-
5	Supination/ plantarflexion	Medial displacement: 1mm	Vertical shear comminuted	Oblique, Weber B	NO	2 Lag screw and neutralization plate	Antiglide plate	-
6	SER 4	Medial displacement: 1mm	Transverse	Oblique, Weber B	Yes	1 Lag screw and neutralization plate	1 screw	-
7	SER 4	Medial displacement: 7mm	Oblique with comminution	Weber B	Yes	1 Lag screw and locking plate	Antiglide plate	-
8	SER 4	Medial displacement: 6 mm	Transverse	Weber B	No	1 Lag screw and locking plate	2 screws	-
9	SER 4	Medial displacement: 0 mm	Transverse with sagittal split	Weber B	Yes	1 lateral plate	2 screws	2 posterior screws
10	SER 4	Medial displacement: 11 mm	Transverse	Weber B	NO	1 locking plate	2 screws	-
11	SER 4	Medial displacement: 2 mm	Transverse	Weber B	NO	2 lag crews and neutralization 1/3 semi plate	1 screw	-
12	SER 4	Medial displacement: 5 mm	Oblique	Weber B	NO	1 lag screw and neutralization 1/3 semi plate	Antiglide plate	-
13	PER 4	Medial displacement: 2 mm	Transverse	Short Oblique	NO	1 bridge plate and 2 sydesmosis screws	-	-
14	SER 4	Medial displacement: 10 mm	Transverse	Weber B	YES	1 lag screw and neutralization plate	2 lag screws	-
15	SAD 1	Medial displacement: 0 mm	Vertical	Nil fracture	NO	-	1 antiglide plate	-
16	SER 4	Medial displacement: 4 mm	Transverse	Weber B	YES	1 lag screw and fibula locking plate	1 lag screw	-
17	PER 1/PA 1	Medial displacement: 3 mm	Oblique	Nil fracture	NO	-	2 screws medially	-
18	SER 4	Medial displacement: 4 mm	Transverse	Weber B	NO	3 lag screws and 1/3 semi neutralization plate	Antiglide plate	-
19	SAD 1	Medial displacement: 1 mm	Vertical	Nil fracture	NO	-	2 screws medially	-
20	PER 1/PA 1	Medial displacement: 13 mm	Transverse	Nil fracture	NO	-	2 screws medially	-
21	SER 4	Medial displacement: 8 mm	Transverse	Weber B	YES	1 lag screw and locking plate	1 screw/1 buried kwire	2 posterior screws
22	SER 4	Medial displacement: 4 mm	Transverse	Weber B	NO	1 lag screw and a neutralization plate	2 screws	-
23	Posterior pilon	Medial displacement: 1 mm	Vertical split	Weber C	YES	1 lateral bridge plate	1 posterior plate	-
24	SER 4	Medial displacement: 5 mm	Transverse	Weber B	NO	1 lag screw and locking plate	2 screws	-
25	PER 4	Medial displacement: 9 mm	Transverse	Weber C	YES	Posterior antiglide plate, 1 syndesmosis screw	2 screws	1 antiglide non-locking plate

Exclusion criteria included paediatric fractures/triplane fractures, pilon fractures where the medial malleolus involved the distal tibial plafond and were not ankle fractures.

None of the unaffected feet had previous injuries/surgery. We assessed and calculated midfoot and first ray stability/instability and compared this with the contralateral feet. We also evaluated the spring ligament thickness using ultrasound of the injured foot compared with non-injured.

The Shapiro-Wilk test was used to determine normality. The paired T-test was used to assess normal data. The Wilcoxon rank test was used to assess non-parametric paired data. A p < 0.05 was considered statistically significant. The Mann-Whitney U test was used for non-paired, non-parametric data.

Assessment of first ray instability

A custom-made ankle foot orthosis (AFO) with a digital calliper scale, based on Klaue’s original device and validated by Coughlin, was used to quantify FRI [14]. Increased FRI with ankle plantarflexion was negated by taking measurements with the ankle in a neutral position. The middle column was held firmly against the orthosis’s plastic base to resist middle column dorsiflexion when applying a dorsal force at the plantar metatarsal head through a cutout within the AFO, until a firm endpoint was reached. A probe in contact with the dorsal metatarsal head linked to a sliding digital calliper allowed dorsal surface metatarsal head readings, negating the effect of fat pad compression that may distort readings taken from the plantar surface. FRI was only assessed in the dorsal sagittal plane. The mean of the three (n=3) readings to the nearest 0.1 mm was recorded. FRI was considered significant with a dorsal translation of 8 mm or more [14].

**Figure 1:**
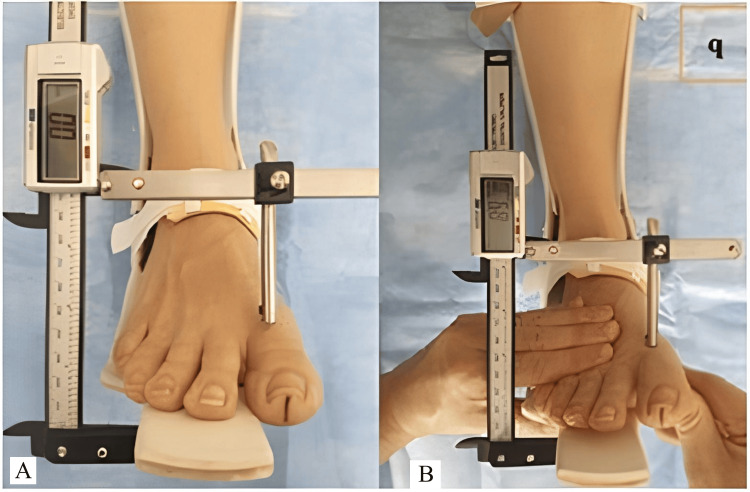
(A) Use of the digital Klauemeter to assess the dorsal sagittal first ray instability. (B) A dorsal probe applied to the metatarsal head with one hand fixing the second metatarsal to the base allows application of a dorsal force with the other hand.

Talonavicular laxity/spring ligament laxity assessment

Lateral translation (LT) of the foot hallux was used as an indirect measure of SL laxity. Previous cadaver studies showed that lateral hallux translation/TN foot abduction was primarily a function of the SL despite tendon loading [12,15,16]. A device where the malleoli, calcaneus and talus were held in a modified padded clamp maintained neutral heel position (no varus/valgus), limited subtalar pronation and decreased hindfoot talus external rotation in the ankle arising from deep deltoid insufficiency. Residual lateral plane abduction laxity occurs mainly due to SL laxity. Greater lateral plane motion represents greater SL laxity. Lateral hallux translation was used as an indirect measure of SL/TN laxity and compared to the uninjured contralateral foot.

**Figure 2:**
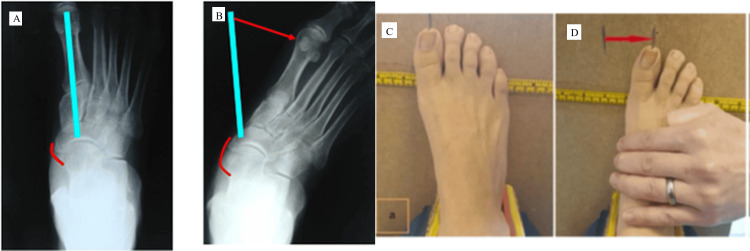
Lateral translation (LT) score assessment. (A) Spring ligament (SL) and the first metatarsal axis. (B) Small strain in the SL can be amplified visually into a large shift at the hallux using the talonavicular axis and the first metatarsal to amplify strain in the SL. (C) The ankle, talus and calcaneum are immobilised in a padded clamp. The second metatarsal is then aligned with the tibial crest (resting foot position). (D) A lateral force is applied to the foot metatarsal head until an end point is reached. The position of the hallux pre and post force application is noted and then measured. SL strain is amplified to lateral forefoot translation which acts as an indirect measure of SL/DSL strain DSL: Deltoid-spring ligament

Leg/tibial rotation was further limited by manually holding the distal tibia, and medial-to-lateral force on the metatarsal head was applied (Figure 2). The second toe was visually aligned with the anterior tibial crest and the centre of the hallux was marked. The forefoot was laterally pushed until a firm endpoint was reached, and the laterally translated position of the hallux was noted. This mimics the neutral heel lateral push test [12]. The mean of the three (n=3) readings to the nearest 1 mm was recorded.

Spring ligament thickness

SL thickness was assessed deep to the tibialis posterior tendon at the level of the sustentaculum tali and thickness was recorded using ultrasound (US) comparing injured vs non-injured feet. 

## Results

Twenty-five patients with paired feet with malleolar fractures who underwent surgical treatment between 2021 and 2022 were identified.

There were 15 left and 10 right injured feet. Twenty-one female patients and four male patients were identified. The average age of the patients at the time of injury was 55.2 years (SD 13.68 years, range 18-76 years) (Table 3). Patients´ average height was 166 cm (SD 8.4 cm; range: 155-183). The average BMI of the patients was 31.04 (SD 5.0, range 24-44.1). The Beighton score showing laxity was 1.3 (SD 1.29: range 0-5). The average foot length was 24.8 (SD 1.59, range 23-30). The average time from injury to clinic was 66.6 months (SD 52.26 months, range 2-60 months). About 67% of the patients suffered SER 4 Lauge Hansen fractures and 33% other types.

**Table 3:** Demographics: patient demographics including age, gender, height, BMI and gender have been included. Data include mean score, SD and the range.

Mean SD (Range)	Subgroup	Value
Female	n = 21 (84.6%)	-
Male	n = 4 (15.4%)	-
Age (years)	55.2 ± 13.68	Range (18 - 76)
Height (cm)	166 ± 8.4	Range (155 - 183)
BMI	31.04 ± 5	Range (24 - 44.1)
Beighton score	1.3 ± 1.29	Range (0 - 5)
Affected Foot Length (cm)	24.8 ± 1.59	Range (23 - 30)
Time Since Injury (weeks)	66.6 ± 52.26	Range (2 - 60)
Type of fracture	SER 4 67%	Others 33%
Laterality of Injury	Left (n)	15
Laterality of Injury	Right (n)	10

Results of mean average measurements of SL thickness, LT and FRI of each patient are noted in Table 4.

**Table 4:** Results of measurements: SL thickness, lateral translation (LT) and first ray instability (FRI) of each patient comparing injured vs non-injured feet.

Patient no	Laterality of injury	SL thickness (US) mean measurement of 3 (cm)		Lateral translation score mean measurement of 3 (mm)		First ray stability score mean measurement of 3 (mm)	
		R	L	Affected side	Unaffected side	Affected side	Unaffected side
								1	Left	0.57cm	0.6cm	56	12	13.1	2.1
2	Left	0.48cm	0.45cm	45.3	14	10.7	3.9
3	Right	0.49cm	0.38cm	47.7	41.3	6.6	3.1
4	Left	0.56cm	0.46cm	62.3	20.7	12.1	4
5	Left	0.49 cm	0.36cm	67.6	27	11.6	11.5
6	Left	0.44cm	0.54cm	67.3	22.3	10.8	3.5
7	Right	0.47cm	0.47cm	50.6	38.7	6.2	5.5
8	Left	0.36cm	0.6cm	48	22.3	13.1	6.6
9	Right	0.45cm	0.32cm	57	37.7	6.4	2.4
10	Left	0.4cm	0.48cm	52.3	15.3	11.9	9
11	Left	0.35cm	0.34cm	60	32	9.5	6
12	Right	0.33cm	0.37cm	65	24.3	6.3	2.9
13	Right	0.33cm	0.33cm	70.3	36.7	10.7	3.7
14	Left	0.35cm	0.33cm	46.3	21.3	9	2.4
								15	Right	0.33cm	0.36cm	22	18	1.7	2.8
16	Left	0.46cm	0.51cm	43.3	22	9.7	5.3								
								17	Right	0.52cm	0.52cm	26.6	26.7	1.4	2.3
18	Right	N/A	N/A	24.6	26	8.7	3.6								
								19	Left	N/A	N/A	51.6	22	7.6	0.9
20	Right	N/A	N/A	25	30.3	5.1	10.2								
21	Right	0.51cm	0.50cm	5.6	20.3	2.5	5.2
								22	Left	0.64cm	0.45cm	53.6	19.7	12.6	5.7
23	Left	0.36cm	0.44cm	59.3	23.7	10	4								
								24	Left	0.42cm	0.65cm	59.3	34	10.9	5.6
25	Left	0.53cm	0.48cm	51.6	12.7	12	4.1								

In 20 patients, the injured foot showed an increased lateral translation score and in 19 patients, an increased tarsometatarsal instability score (48.74 ± 16.34, range 5.6-70.3) when compared to the uninjured foot (25.56 ±7.85, range 3.1 - 38.6). This difference was statistically significant with a p-value (p < 0.05: data not normal: Wilcoxon rank test) (see Appendices).

There was a statistically significant difference in FRI scores between affected (9.07 ± 3.10 range 0.8 - 14.4) and unaffected sides (4.64 ± 2.46 range 0.7 - 12.1) with p-value p<0.01. There was no difference in SL thickness p=0.78 (p>0.05: normal data: paired t-test) between affected (0.47 ± 0.10 range 0.33 - 0.65) and unaffected feet (0.44 ± 0.09 range 0.32 - 0.64) (Table 5). Three patients did not have SL thickness measurements as they refused a scan.

**Table 5:** Medial arch instability. Mean, standard deviation and range of the lateral translation scores and p-value were noted.

Variable	Affected side	Unaffected side	Wilcoxon-rank test (non-parametric)/ T test for parametric data	Z/T score
First ray instability score (mm)	9.07 ± 3.2 (0.8 – 14.4)	4.7 ± 2.5 (0.9 – 11.5)	p < 0.05	z = -3.7132
Lateral translation score (mm)	48.74 ± 16.3 (5.6 – 70.3)	24.1 ± 8.46 (3.1 – 38.6)	p < 0.05	z = -3.9957
Ultrasound thickness of SL (mm)	0.47 ± 0.10 (0.33 – 0.65)	0.44 ± 0.09 (0.32 – 0.64)	p > 0.05	t = -0.946246

In the group of rotational mechanisms of fractures, we divided into the SER 4 group (n=16) and PER/PA group (n=5). Specifically, in the SER 4 group, there was a significant difference in LT and FRI scores, p< 0.05. The pronation injury group did not demonstrate a statistically significant increase in the lateral translation scores between affected and unaffected feet (p>0.05; see Table 6). However, three of the pronation injuries (PER 1 and PER 4) did demonstrate measurements of instability (patient numbers 2, 13 and 25).

**Table 6:** Rotational injuries demonstrated increased lateral translation scores compared to the unaffected side.

Injury type	Lateral translation score, affected (mm)	Lateral translation score, unaffected (mm)	p-value	Z/T score	First ray instability score, affected (mm)	First ray instability score, unaffected (mm)	p-value	Z/T score
SER 4 (n = 16)	52.6 ± 14.6	25.7 ± 8.6	P < 0.05	z = -3.3611	9.52 ± 3.1	5.1 ± 2.5	P < 0.05	t = -5.317266
PER 1–4/PA3 (n = 5)	43.8 ± 18.8 (25–70.3)	24 ± 10.4	P > 0.05	T = -2.126203	7.98 ± 4.5	4.8 ± 3.1	p > 0.05	t = -1.207817

In four non-rotational injuries (three supination adduction injuries and one posterior pilon injury), affected feet also had a mean increase in lateral translation scores and FRI scores greater than 20 mm in both measurements, but without statistical significance (p>0.05) (Table 7).

**Table 7:** Non-rotational injuries demonstrating increased lateral translation scores and first ray instability compared to the non-affected side although not significant.

Injury type	Lateral translation score, affected (mm)	Lateral translation score, unaffected (mm)	p-value	T score	First ray instability score, affected (mm)	First ray instability score, unaffected (mm)	p-value	T score
Supination–adduction (n: 3)/Posterior pilon (n: 1)	39.38 ± 18.9	22.4 ± 3.7	p > 0.05	-1.8455	8.65 ± 1.0	2.82 ± 1.4	p > 0.05	-2.3343

Finally, in our analysis of the association between lateral translation scores of affected feet with medial malleolus displacement, 17/25 patients had more than or equal to 2 mm displacement as measured using X-rays/radiographs taken prior to surgery. Lateral translation scores measuring laxity in affected feet, with less or equal then to 2 mm medial malleolus displacement (eight patients; mean 56.89 mm and SD 15.72) compared to medial malleolus fractures with greater than 2 mm displacement (17 patients; mean 44.89 mm and SD 15.41) were statistically significantly different (p < 0.05, non-parametric: Mann-Whitney U test), although surprisingly, the absolute values in the less displaced groups had higher scores. Therefore, displacement of the medial malleolus was not a predictor for the development of increased TN laxity (Table 8).

**Table 8:** Association between lateral translation scores of affected feet and medial malleolus displacement.

Medial malleolus displacement prior to surgery	Lateral translation score of the affected feet (mm, Mean ± SD)
≤ 2 mm displacement (n = 8)	56.89 ± 15.72
≥ 2 mm displacement (n = 17)	44.89 ± 15.41
p-value (two-tailed t-test)	> 0.05

## Discussion

The effect of medial malleolus fractures on the development of midfoot laxity has not been extensively studied. Surgical prioritisation of the bony components often means the assessment or intraoperative reconstitution of superficial deltoid/SL laxity is not performed. This potentially sets the foot up for secondary destabilisation of the first ray.

Superficial deltoid has a 1.3 cm origin at the malleolus and its wide origin means fibres can be disrupted from a medial malleolus fracture [17]. It is known that medial malleolus fractures can disrupt the fibres of the deep DL. Fukuyama et al. [11] demonstrated that in 50% of supination-external rotation ankle injuries, the medial malleolus fracture disrupts the deep deltoid fibres. The size of the medial fragment injury is closely linked to the competence and integrity of the DL. Tornetta [17] found that medial malleolus fractures greater than 2.8 cm in length (supracollicular fractures) preserve the integrity of the deep DL, while fractures smaller than 1.7 cm (such as fractures of the anterior colliculus or intercollicular fractures) compromise the DL.

Amaha’s and Apoorva's dissection studies [18,19] independently demonstrated the internal morphology of the combined deltoid-spring ligament (DSL) complex, which consists of the superficial deltoid and SL as a single continuous structure that can be reflected as one from the medial malleolus. Inferiorly, the region known as the SL contains a fibrocartilaginous area. Pankovich classified the ligament into segments: the anterior segment consists of portions of the SDL attached to the navicular; the middle segment comprises fibres connected to the ST fragment; and the posterior fibres form the posterior segment. The TN axis has no additional intrinsic ligaments influencing it medially, making it more vulnerable to laxity and thus a more reliable indicator for assessing dysfunction of the superficial deltoid/SL using clinical tests such as the Neutral Heel Lateral Push (NHLP) test [12]. The anteromedial draw test and the heel external rotation test, which apply rotational and anterior translation forces to the tibiotalar joint, can concomitantly assess for deep DL laxity. Both the superficial and deep DLs function as primary restraints against pronation [20,21].

Despite the morphological and band variability of the superficial DL fibres [20], they are often disrupted in cases of medial malleolar fractures [22-25]. A significant portion of the superficial deltoid originates from the anterior colliculus, meaning that even small fractures of the anterior medial malleolus can damage the tibiospring fibres.

Several biomechanical studies have evaluated the DSL. The tibiotalar and tibiocalcaneal fibres prevent tibiotalar valgus angulation. Tibiospring not only resists TN abduction [26,27] but can also affect the stability of the ankle joint. The tibiocalcaneal ligament is most strained in pronation [23,28] and foot dorsiflexion, particularly during midstance weight-bearing. This tightening of the tibiocalcaneal portion with ankle dorsiflexion [16] may make it more susceptible to forces, as it may already be at the edge of its elastic limit.

Secondary factors that may further weaken the DSL include strain with rotational/ pronatory injury forces that arise with cyclical foot loading, exacerbating TN laxity.

Disruption of the DSL can progress normal feet into stage 0 flatfoot [13]. Deep deltoid disruption has been shown to result in FRI and TN abduction laxity in vivo, within 3 to 6 months of loading [10]. The absence of an intact SL forces the stable first ray to act as an antipronator, eventually leading to its failure, as demonstrated in Chu’s cyclical loading cadaver study [29]. Jennings demonstrated that isolated SL sectioning can lead to peritalar changes associated with flatfoot [15].

The rotational and abduction forces leading to an avulsion fracture of the medial malleolus in PER 1-4/PA 1 and SER 4 injuries may strain the superficial DL prior to fracture, especially in younger patients where bone tensile strength may be higher. In PE rotation injuries, the zone of injury typically starts medially, resulting in greater strain on the SL and medial deltoid. The same forces that cause an avulsion of the medial malleolus in these injuries could also strain the DSL.

In supination external rotation injuries, foot supination with external rotation theoretically provides greater protection to the SL from strain by locking Chopart's joint to resist TN abduction during ankle external rotation. Despite this, we found no significant difference in FRI and lateral translation scores between SER 4 and pronation injuries. We acknowledge that a larger sample size may be necessary to detect any true differences.

In our study, three patients were identified as having injuries that did not result in an abduction or external rotation force that could strain the SL component of the DSL. In fact, forces in these injuries act in a direction that offloads the SL and other medial soft tissue structures [24]. Three patients had supination adduction injuries, with one not developing any increased medial column instability (no increase in LT scores or FRI scores). One patient had a posterior pilon fracture resulting in a coronal medial malleolus split. All fractures had excellent mortise reduction/fixation (reduced to less than 1 mm displacement). SL strain/TN abduction laxity most likely arises secondarily to capsule/DSL fibre disruption from the fracture and the secondary strain from cyclical foot loading, but not necessarily from the mechanism of force injury. All fractures had excellent mortise reduction/fixation (reduced to less than 1 mm displacement of the medial malleolus). The coronal split that developed increased TN laxity despite minimal disruption to the malleolus and having a posterior plating which would have kept the anterior and medial deltoid attachments/components free from surgical insult. SL strain/TN abduction laxity in this fracture most likely arose secondarily to more subtle DSL fibre disruption from the fracture and the secondary strain from cyclical foot loading, but not necessarily from the mechanism of force injury.

Dysfunction of the SL portion may occur from the superficial deltoid, given similar US findings in injured vs non injured group. Superficial fibre disruption rather than intrinsic SL strain [30] may be causal. SL thickness therefore may not reflect TN abduction laxity. Furthermore, displacement of the medial malleolus does not reflect the extent of TN instability. Our results demonstrated a greater amount of TN instability in patients with less than 2mm translation (see Table 7). Reasons may reflect our small sample size or static radiographs may not represent true displacement, where the injury displacement may have been greater. However, our results demonstrate that radiographic displacement does not correlate with the extent of soft tissue compromise/TN laxity postoperatively, although our result was not significant.

These results align with those of Stufkens et al., who demonstrated that only 21.7% of optimally reduced fractures had poorer long-term outcomes [5]. When considering the treatment of ankle fractures involving the medial malleolus is not just isolated to the ankle joint but has broader implications on wider foot dysfunction. Prolonged protection of the medial arch in ankle fractures involving the medial malleolus, using orthotics and prognostic counselling about the broader foot dysfunction and arch instability injury, may be required. Surgical intervention aimed at restoring superficial DL function could improve TN abduction stability and overall foot function. Hinterman reported 90% positive outcomes in patients undergoing superficial DL repair [28], which aligns with a meta-analysis assessing 192 patients [31]. Repairing the DSL function resists medial talar head subluxation and prevents secondary destabilisation of the first ray. Previous studies have mainly explored medial instability in the context of DL rupture, whereas instability secondary to medial malleolus fractures has received little direct investigation.

Limitations

This study does not isolate the effect of the original medial malleolus fracture from any surgical disruption deltospring ligament. This can only be assessed in patients with isolated medial malleolus fractures treated non-operatively. The role of MRI in assessing soft tissue disruption was not evaluated. CT scans were not available for all fractures. Assessing retromalleolar tenderness involves subjectivity. TN coverage on weight-bearing AP radiographs could have provided additional objective support for the observed differences in TN instability. The small sample size (n = 25) is a limitation, as it affects the statistical power and generalisability of the findings.

## Conclusions

Medial malleolus fractures can indicate broader medial column and midfoot instability, not just isolated ankle injury. Even small or minimally displaced fractures may disrupt the DSL complex, leading to TN laxity and secondary FRI. Postoperative instability is poorly predicted by fracture displacement or fragment size and may occur despite excellent mortise reduction, reflecting unrecognised ligament injury and cyclical loading.

Careful assessment of medial ligament function and midfoot stability, along with prolonged protection of the medial arch, may be warranted, especially in younger, active patients. Future studies using advanced imaging and non-operative cohorts are needed to guide evidence-based management of ligamentous injury associated with medial malleolus fractures.

## Appendices

**Table 9:** Raw data - lateral translation score.

Patient No.		Affected side				Unaffected side		
1		62	55	51		12	11	13
2		44	46	53		45	42	37
3		67	60	60		16	23	23
4		67	68	66		27	27	27
5		65	70	67		22	22	23
6		53	59	60		41	39	36
7		50	50	49		21	22	24
8		55	55	61		39	37	37
9		53	54	50		15	17	14
10		64	59	57		30	30	36
11		63	65	67		25	24	24
12		48	46	45		22	20	22
13		45	42	43		20	21	25
14		8	1	8		22	20	19
15		25	25	24		29	26	27
16		53	52	50		12	12	14
17		61	60	57		31	34	37
18		68	71	72		36	36	38
19		54	53	54		19	18	22
20		41	47	48		13	14	15
21		22	24	29		29	30	32
22		23	27	30		25	27	28
23		55	49	51		22	20	24
24		21	22	23		19	17	15
25		64	60	54		22	23	26

**Table 10:** Raw data - first ray instability score.

Patient No.		Affected side				Unaffected side		
1		10.5	14.4	14.3		1.6	1.3	3.3
2		6.7	5.5	7.7		3.2	1.8	4
3		12.8	8.1	12.1		4.5	4	3.5
4		11.5	13	10.3		12.1	11.3	11.1
5		10.8	10.6	11.1		3.9	3.7	2.8
6		6.7	6	6		5.1	4.8	6.7
7		12.3	12.6	13.1		7.7	5.7	6.4
8		6.9	6.2	6		3.5	2.3	1.5
9		12.7	10	12.9		8.3	9.2	9.5
10		10.4	9.2	8.9		5.8	6.1	6
11		6.8	5.3	6.7		2.9	3.1	2.7
12		6.8	8.8	11.3		3	2.4	1.9
13		8.9	8.9	11.2		5.5	6.3	4.2
14		2.4	2.3	2.8		4.9	7.3	3.4
15		8.9	8.6	8.6		3.2	3.8	3.9
16		13.2	12.9	9.8		3.6	3.6	2.4
17		10.1	13.4	9.11		4.7	6.1	6
18		10.4	11.7	9.9		4	2.9	4.2
19		13.7	12.5	11.5		5.2	4.7	7.1
20		9.2	9.9	13.1		4	3.5	4.3
21		5	4.3	6.1		9.4	10.9	-
22		1.2	1.7	1.3		2.7	2.3	1.8
23		5.6	8.7	8.5		0.7	0.9	1.1
24		0.8	1.9	2.1		1.9	3.1	3.3
25		9.5	10.1	10.6		5.4	4	2.7
